# C9orf142 transcriptionally activates MTBP to drive progression and resistance to CDK4/6 inhibitor in triple‐negative breast cancer

**DOI:** 10.1002/ctm2.1480

**Published:** 2023-11-27

**Authors:** Li Liao, Ling Deng, Yin‐Ling Zhang, Shao‐Ying Yang, Lisa Andriani, Shu‐Yuan Hu, Fang‐Lin Zhang, Zhi‐Min Shao, Da‐Qiang Li

**Affiliations:** ^1^ Fudan University Shanghai Cancer Center and Institutes of Biomedical Sciences Fudan University Shanghai China; ^2^ Cancer Institute, Shanghai Medical College Fudan University Shanghai China; ^3^ Department of Oncology, Shanghai Medical College Fudan University Shanghai China; ^4^ Department of Breast Surgery, Fudan University Shanghai Cancer Center Fudan University Shanghai China; ^5^ Shanghai Key Laboratory of Breast Cancer, Shanghai Medical College Fudan University Shanghai China; ^6^ Shanghai Key Laboratory of Radiation Oncology, Shanghai Medical College Fudan University Shanghai China

**Keywords:** C9orf142, cancer progression, CDK4/6 inhibitor, MTBP, triple‐negative breast cancer

## Abstract

**Background:**

Triple‐negative breast cancer (TNBC) presents the most challenging subtype of all breast cancers because of its aggressive clinical phenotypes and absence of viable therapy targets. In order to identify effective molecular targets for treating patients with TNBC, we conducted an integration analysis of our recently published TNBC dataset of quantitative proteomics and RNA‐Sequencing, and found the abnormal upregulation of chromosome 9 open reading frame 142 (C9orf142) in TNBC. However, the functional roles of C9orf142 in TNBC are unclear.

**Methods:**

In vitro and in vivo functional experiments were performed to assess potential roles of C9orf142 in TNBC. Immunoblotting, real‐time quantitative polymerase chain reaction (RT‐qPCR), and immunofluorescent staining were used to investigate the expression levels of C9orf142 and its downstream molecules. The molecular mechanisms underlying C9orf142‐regulated mouse double minute 2 (MDM2)‐binding protein (MTBP) were determined by chromatin immunoprecipitation (ChIP) and dual‐luciferase reporter assays.

**Results:**

In TNBC tissues and metastatic lymph nodes, we observed that C9orf142 exhibited abnormal up‐regulation, and its elevated expression was indicative of unfavorable prognosis for TNBC patients. Both in vitro and in vivo functional experiments demonstrated that C9orf142 accelerated TNBC growth and metastasis. Further mechanism exploration revealed that C9orf142 transcriptionally activated MTBP, thereby regulating its downstream MDM2/p53/p21 signaling axis and the transition of cell cycle from G1 to S phase. Functional rescue experiment demonstrated that knockdown of MTBP attenuated C9orf142‐mediated tumour growth and metastasis. Furthermore, depletion of C9orf142 remarkably increased the responsiveness of TNBC cells to CDK4/6 inhibitor abemaciclib.

**Conclusions:**

Together, these findings unveil a previously unrecognized effect of C9orf142 in TNBC progression and responsiveness to CDK4/6 inhibitor, and emphasize C9orf142 as a promising intervention target for TNBC treatment.

## Graphical abstract

1. C9orf142 is elevated in TNBC patients and its elevation predicts unfavourable clinical outcome.

2. C9orf142 augments TNBC progression through transcriptional transaction of MTBP to regulate its downstream MDM2/p53/p21 signaling axis. 3. C9orf142 promotes resistance of TNBC cells to CDK4/6 inhibitor partially depend on MTBP/MDM2/p53/p21 signaling axis.

4. Knockdown of C9orf142 inhibits TNBC progression and enhances its sensitivity to CDK4/6 inhibitor.

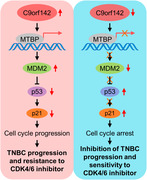

## 1 INTRODUCTION

Approximately 15%−20% of breast cancers are classified as triple‐negative breast cancer (TNBC).^1^ In comparison to other types of breast cancer, TNBC exhibits the most aggressive clinical characteristics, including a younger age of onset, a higher incidence of early recurrence and distant metastasis, a lower 5‐year survival rate, and a lack of established therapeutic targets.^1^, ^2^ Due to the negative status of hormone receptors and human epidermal growth factor 2 (HER2), TNBC patients do not benefit from well‐established endocrine and HER2‐targeted therapies. Thus, chemotherapy serves as the primary systemic treatment choice for patients with TNBC, but the development of drug resistance ultimately results in the failure of treatment.^3^ In order to address this challenge, numerous innovative focused substances have emerged in recent years, including inhibitors of cyclin‐dependent kinases 4 and 6 (CDK4/6), which are being actively investigated in preclinical studies and clinical trials as possible treatments for TNBC in addition to hormone receptor‐positive breast cancer.^4^, ^5^, ^6^, ^7^, ^8^ The CDK4/6 inhibitors arrest the cell cycle through the G1/S checkpoint.^9^ Additionally, CDK4/6 antagonists, including palbociclib, abemaciclib, and ribociclib, have been approved by the U.S. Food and Drug Administration (FDA) for the treatment of various types of breast cancer.^10^ However, the determinants of responsiveness to CDK4/6 inhibition in TNBC are poorly understood.

Chromosome 9 open reading frame 142 (C9orf142), also known as PAXX (paralog of XRCC4 and XLF), is a newly identified member of the X‐ray repair cross complementing 4 (XRCC4) family and participates in repairing damaged DNA through the non‐homologous end joining (NHEJ) pathway.^11^, ^12^, ^13^ In this context, C9orf142 interacts directly with the Ku70‐Ku80 heterodimer and stabilizes NHEJ proteins on damaged chromatin in cells.^11^ Evidence from mouse model studies indicates that C9orf142 is a compensatory factor in the development of central nervous and immune systems,^14^, ^15^ and that combined deficiency of C9orf142 and XRCC4‐like factor (XLF) results in embryonic lethality.^16^, ^17^, ^18^ Interestingly, it was recently documented that C9orf142 is highly expressed in colon cancer tissues due to its promoter hypo‐methylation and acts as an independent prognosticator in colon cancer.^19^ In addition, C9orf142 is up‐regulated in chemoresistant osteosarcoma^20^ and glioma^21^ cells. Interestingly, examination of our recently released TNBC proteomic^22^ and transcriptomic^23^ datasets reveals that C9orf142 is markedly increased in TNBC tissues and has a positive correlation with lymph node metastasis of TNBC patients. Despite these preliminary observations, the precise biological functions and related mechanisms of C9orf142 in cancer progression and therapeutic responsiveness still remain mysterious.

MDM2‐binding protein (MTBP) was initially discovered as a binding partner of MDM2 by a yeast two‐hybrid screen.^24^ Subsequent studies demonstrate that MTBP stabilizes MDM2 through inhibiting its auto‐ubiquitination and promotes MDM2‐mediated degradation of p53 and E‐cadherin.^25^, ^26^ Notably, MTBP is highly expressed in TNBC patients and plays a critical role in promoting tumour growth, survival and resistance to anticancer agents.^27^, ^28^, ^29^, ^30^ The regulation of MTBP in human cancer, despite its functional significance, remains largely unclear.

In this study, we present the initial proof that C9orf142 facilitates the advancement and resistance to abemaciclib, a CDK4/6 inhibitor, in TNBC. Mechanistic investigations revealed that C9orf142 augments TNBC progression through transcriptional transaction of MTBP to regulate its downstream MDM2/p53/p21 signaling axis. Collectively, these discoveries reveal a previously undisclosed biological role and regulatory mechanism of C9orf142 in the advancement of TNBC and its resistance to CDK4/6 inhibitors, highlighting C9orf142 as a potential target for therapeutic intervention in TNBC patients.

## 2 MATERIALS AND METHODS

### 2.1 Clinical data and samples

The recently published expression profiles of quantitative proteomic dataset (normal samples: *n* = 72; TNBC samples: *n* = 90)^22^ and RNA‐sequencing (RNA‐Seq) dataset (normal samples: *n* = 88; TNBC samples: *n* = 360)^23^ were obtained from Fudan University Shanghai Cancer Center (FUSCC) TNBC cohort. The tissues used in this study were collected from TNBC patients who underwent surgery in FUSCC. The FUSCC Ethics Committee approved all experimental procedures.

### 2.2 Cell culture

We obtained HMEC and MCF10A cell lines, which are normal human mammary epithelial cells, as well as LM2‐4175, HCC1806, SUM159, BT549, HCC1937, MDA‐MB‐231 and Hs578T cell lines, which are human TNBC cells used in this study from Shanghai Key Laboratory of Breast Cancer. The above cells were cultured in medium as described previously.^31^

### 2.3 DNA plasmids and vector transfection

The cDNA was subcloned into the pCDH‐CMV‐3× Flag‐Puro vectors. The specific short hairpin RNA (shRNA) sequences targeting C9orf142 and MTBP were acquired from Sigma‐Aldrich Advanced Genomics (www.sigmaaldrich.cn) (Table S1). The primer sequences were synthesized by HuaGene Biotech. The primers were subsequently annealed and then inserted into the pLKO.1 vectors. The complete human C9orf142 cDNA was amplified using the primers specfied in Table S2. Vector transfection, lentivirus packaging, infection and generation of stable cell lines were carried out according to the methods outlined in previous studies.^32^, ^33^

### 2.4 LC‐MS/MS analysis

Liguid chromatograph tandem mass spectrometry (LC‐MS/MS) analysis was conducted in the Institutes of Biomedical Sciences, Fudan University as described previously.^32^ For analyzing label‐free quantitative proteomic results, the fold change of 1.5‐fold was established as the threshold for differentially expressed proteins, requiring at least two unique peptides.

### 2.5 Immunoblotting assays

Proteins were lysed and quantified as previously described.^32^ A total of 20−50‐μg proteins, isolated from cells or tissues, were separated using sodium dodecyl sulfate‐polyacrylamide gel electrophoresis (SDS‐PAGE) and transferred onto polyvinylidene fluoride (PVDF) membranes. The detailed information of the primary antibodies is provided in Table S3. The protein signals were detected using an enhanced chemiluminescence (ECL) kit (Tanon, No. 180−5001E) after incubation with corresponding secondary antibodies.

### 2.6 RT‐qPCR assays

Total RNA extraction, cDNA synthesis, removal of genomic DNA, and real time‐quantitative PCR (RT‐qPCR) assays were carried out according to the methods outlined in previous studies.^32^ Detailed information of the primers utilized for RT‐qPCR in this investigation is provided in Table S4.

### 2.7 Cell proliferation, colony formation and flow cytometry assays

Briefly, for cell proliferation assays, cells were seeded into 96‐well plates in triplicate. Subsequently, they were exposed to either DMSO or abemaciclib (Sellect, No. S5716) at the specified concentrations. Cell viability was assessed using cell counting kit‐8 (CCK‐8) kit. For colony formation assays, in short, cells were seeded into 6‐well plates in triplicate. Subsequently, they were treated with either DMSO or abemaciclib at the indicated concentrations. After fixed with paraformaldehyde, the survival colonies clones were stained with crystal violet. Flow cytometry assay was used to determine cell cycle, following the previously described protocol.^32^

### 2.8 Cell migration and invasion assays

Cells were re‐suspended in 200 μL of complete medium and then seeded into Transwell inserts without Matrigel or coated with Matrigel for cell migration and invasion assays, respectively. Twenty four‐well plates were filled with 800 μL of complete medium. After 20–24 h of incubation, the migrated and invaded cells attached to the subventricular layer of Transwell inserts were fixed using methanol and stained with crystal violet.

### 2.9 Xenograft tumour experiments

All animal experiments were approved by the Ethics Committee of Fudan University Shanghai Cancer Center (FUSCC) on July 9^th^, 2021, and the registration number of the ethics is No. FUSCC‐IACUC‐2021383. The animals using in this study were all 6‐week‐old female BALB/c nude mice. For xenograft tumour experiments, cells were inoculated into the mammary fat pads of mice. Tumour size was measured every 2 days, and tumour volume was calculated using the method described previously.^32^ To determine the effects of CDK4/6 inhibitor abemaciclib on xenograft tumour growth, phosphate buffered solution (PBS) or abemaciclib (25 mg/kg) was administered by oral gavage daily when the average tumour volumes reached 100 mm^3^. For spontaneous metastasis experiments, cells were inoculated into the mammary adipose tissues of mice. For experimental lung metastasis experiments, cells were injected into the caudal veins of mice. Tumour weight and physical condition of mice were monitored every week. Nude mice were sacrificed once they lost weight, developed dysphagia or cachexia. For experimental axillary lymph node metastasis, cells were inoculated into the mammary adipose tissues of mice. Nude mice were sacrificed once tumour volumes reached 2000 mm^3^ or the maximum diameter of the tumour reached 15 mm.

### 2.10 Chromatin immunoprecipitation assays

Chromatin immunoprecipitation (ChIP) assays were performed using Simple ChIP kit (Cell Signaling Technology, No. 9003). Histone H3 antibody was served as a positive control, whereas normal IgG antibody was employed as a negative control. The primers used for ChIP‐PCR in this investigation are provided in Table S4.

### 2.11 Dual‐luciferase reporter assays

MTBP promoters (four sections ranging from −2000 bp before the transcription start site to 100 bp after it) were cloned into the pGL3 vector to generate pGL3‐MTBP. The primers used to clone pGL3‐MTBP plasmids are provided in Table S5. Subsequently, HEK293T cells were transfected with 50 ng of pRL‐TK Renilla luciferase expression vector and 1 μg of either pGL3 basic vector or pGL3‐MTBP plasmids, following the previously described protocol.^34^ Luciferase assays were conducted with the Dual Luciferase Reporter kit (Yeasen, number: 11402ES60) after transfection for 48 h.

### 2.12 Immunofluorescent staining assays

Cells were re‐suspended in 300 μL of complete medium and then seeded into 24‐well plates with cover glass. The next day, cells were fixed with 4% paraformaldehyde. Permeabilization, antibody incubation and imaging were performed as described previously.^32^, ^33^

### 2.13 Statistical analysis

Statistical analyses of two group were conducted with unpaired Student's *t* tests or one‐way analysis of variance (ANOVA). The log‐rank test was utilized to calculate the survival curve of Kaplan‐Meier plot. The statistical analyses were performed using R software, GraphPad and SPSS. The *p* value less than 0.05 was deemed statistically significant.

## 3 RESULTS

### 3.1 C9orf142 is aberrantly up‐regulated in TNBC tissues and its elevated expression levels are indicatives of poor prognosis for TNBC patients

**FIGURE 1:**
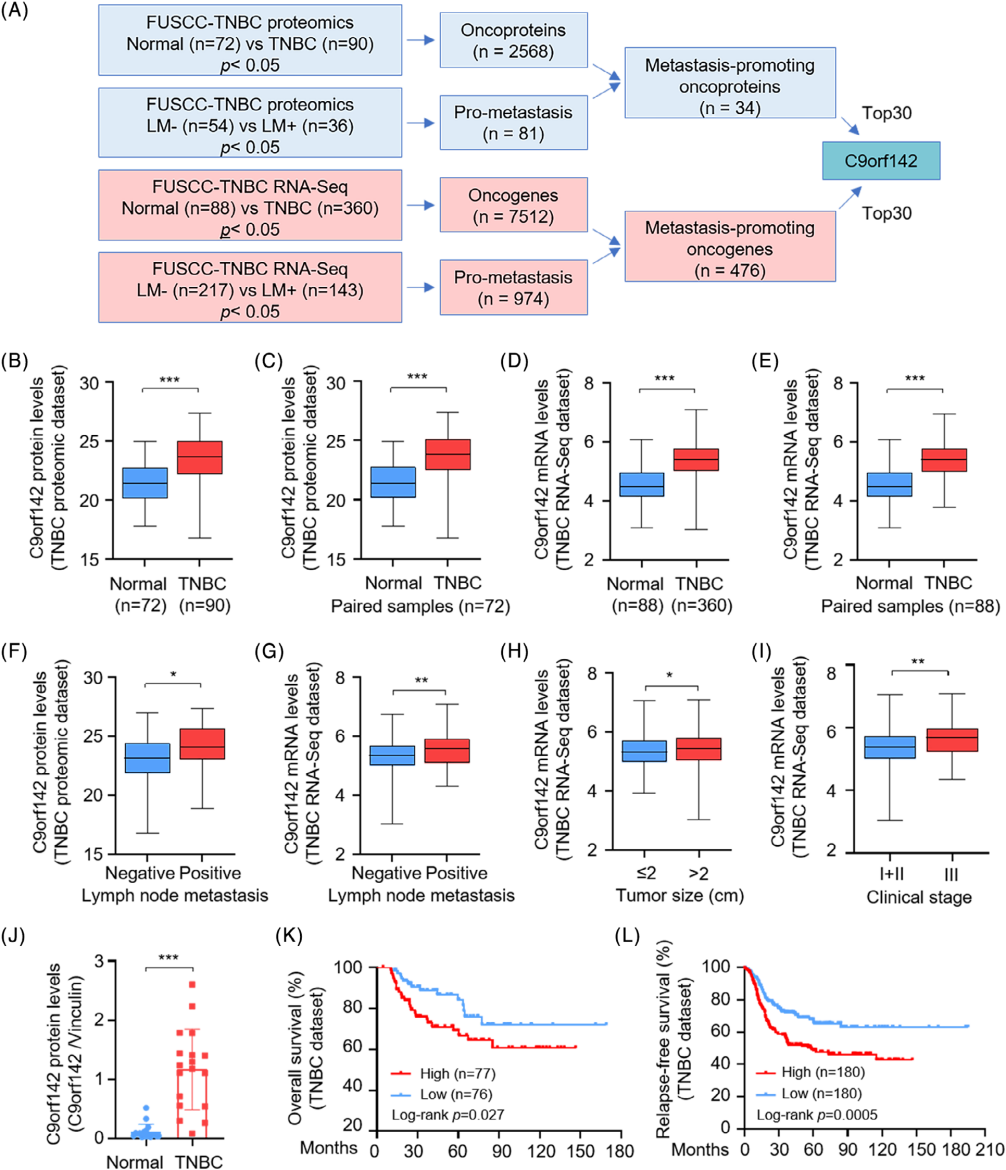
C9orf142 is aberrantly up‐regulated in TNBC tissues and its high expression predicts poor prognosis of TNBC patients. (A) Schematic diagram depicting the screening of metastasis‐promoting oncogenes using the quantitative proteomic dataset and RNA‐Seq dataset from Fudan University Shanghai Cancer Center (FUSCC)‐TNBC project cohort. LM, lymph node metastasis. (B and C) Protein expression levels of C9orf142 in 90 TNBC tissues and 72 adjacent normal tissues in the FUSCC‐TNBC proteomic dataset. (D and E) C9orf142 mRNA expression levels in 360 TNBC tissues and 88 adjacent normal tissues in the FUSCC‐TNBC RNA‐Seq dataset. (F) Protein expression levels of C9orf142 in TNBC tissues with or without lymph node metastasis. (G) C9orf142 mRNA levels in TNBC tissues with or without lymph node metastasis. (H and I) Correlation between C9orf142 mRNA levels and tumour size (H) as well as clinical stage (I). (J) Quantitative results of C9orf142 protein expression levels in 18 pairs of TNBC tissues and matched normal breast tissues. Corresponding immunoblotting images are shown in Figure S1F. (K and L) Kaplan–Meier analysis of the overall survival (K) and relapse‐free survival (L) of TNBC patients with high or low expression levels of C9orf142 using Kaplan–Meier Plotter database (http://kmplot.com/analysis/index.php?p = service). *p < 0.05; **p < 0.01; ***p < 0.001; ns, no significance.

In order to identify possible therapeutic targets that promote the progression of TNBC, we initially conducted an integration analysis of both TNBC proteomic^22^ and transcriptomic dataset^23^ from our center project cohort (Figure 1A). A total of 2568 proteins in the proteomic dataset were abnormally increased in TNBC samples when compared to adjacent normal samples. According to the status of lymph node metastasis, we found that 81 proteins were upregulated in TNBC patients with lymph node metastasis (*n* = 36) relative to those without lymph node metastasis (*n* = 54). Further examination revealed that 34 proteins were elevated in both TNBC patients and those with lymph node metastasis (Figure 1A, upper panel). Similarly, a total of 7512 genes in RNA‐Seq dataset were overexpressed in TNBC tissues as compared with normal tissues. Moreover, 974 genes were elevated in patients with lymph node metastasis (*n* = 143) relative to those without lymph node metastasis (*n* = 217). Further analysis demonstrated that a total of 476 oncogenes were positively correlated with lymph node metastasis of TNBC patients (Figure 1A, lower panel). According to the *p* value, the top 30 metastasis‐related proteins and genes are presented in Figures S1A–D.

Interestingly, we noticed that C9orf142 was upregulated in total and paired TNBC specimens in both the quantitative proteomic dataset (Figure 1B,C) and the RNA‐Seq dataset (Figure 1D,E). Moreover, C9orf142 expression was higher in TNBC patients with lymph node metastasis than those without lymph node metastasis (Figure 1F,G) and was positively correlated with tumour size (Figure 1H) and clinical stage (Figure 1I) of TNBC patients. Consistently, analysis of the TCGA breast cancer database also demonstrated that C9orf142 was upregulated in TNBC samples, and its expression levels in TNBC were slightly higher than that in luminal and HER2‐positive subtypes of breast cancer (Figure S1E). To validate the above results, we detected the protein expression levels of C9orf142 in paired TNBC tissues. Results showed that in comparison with the normal counterparts, C9orf142 exhibited a notable increase in 83.3% (15/18) TNBC tissues (Figure 1J and Figure S1F). Analysis of survival using Kaplan–Meier Plotter database revealed that the difference of C9orf142 expression was not related to the overall survival and relapse‐free survival prognosis of all types of breast cancer patients (Figures S1G,H). However, increased C9orf142 expression correlated with worse outcomes in TNBC patients, including overall survival (OS) and relapse‐free survival (RFS), suggesting that C9orf142 may serve as a prognostic factor for TNBC patients (Figure 1K,L). Taken together, these findings suggest that C9orf142 could function as a novel oncogene to drive the advancement of TNBC, and its elevated levels are indicatives of unfavorable prognosis in individuals with TNBC.

### 3.2 C9orf142 promotes TNBC growth both in vitro and in vivo

**FIGURE 2:**
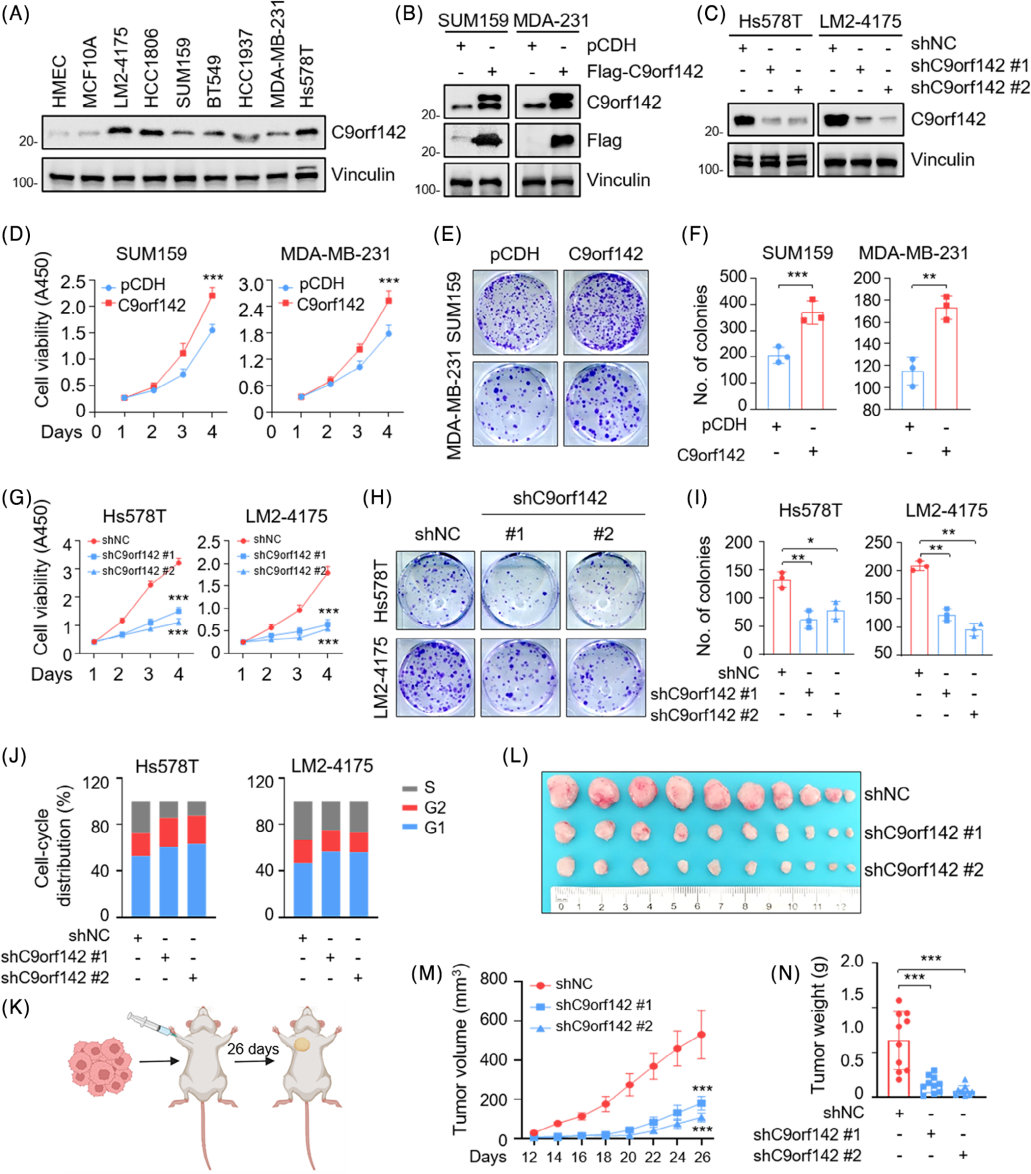
C9orf142 promotes TNBC cell growth both in vitro and in vivo. (A) Immunoblotting analysis of C9orf142 protein expression levels in two human mammary epithelial cell lines and seven human TNBC cell lines. (B and C) Immunoblotting analysis of C9orf142 expression status in SUM159 and MDA‐MB‐231 cells stably expressing empty vector pCDH or Flag‐C9orf142 (B) and in Hs578T and LM2‐4175 cells stably expressing empty vector shNC or shC9orf142 (#1 and #2) (C). (D–F) SUM159 and MDA‐MB‐231 cells stably expressing empty vector pCDH or Flag‐C9orf142 were subjected to CCK‐8 (D) and colony formation assays (E–F). Representative images of survival colonies (E) and corresponding quantitative results (F) are shown. (G–I) Hs578T and LM2‐4175 cells stably expressing empty vector shNC or shC9orf142 (#1 and #2) were subjected to CCK‐8 (G) and colony formation assays (H–I). Representative images of survival colonies (H) and corresponding quantitative results (I) are shown. (J) Flow cytometry analysis of cell‐cycle distribution of Hs578T or LM2‐4175 cells stably expressing empty vector shNC and shC9orf142 (#1 and #2). (K–N) A total of 1 × 10^6^ LM2‐4175 cells stably expressing shNC or shC9orf142 (#1 and #2) were inoculated into mammary fat pads of 6‐week‐old BALB/c female nude mice (*n* = 10). After 26 days of injection, mice were sacrificed and xenograft tumours were removed. Schematic diagram depicting experimental procedure (K), image of removed xenograft tumours (L), tumour volume (M) and tumour weight (N) are shown. Panel K was created with BioRender.com. *p < 0.05; **p < 0.01; ***p < 0.001; ns, no significance.

In order to verify the possible functions of C9orf142 in the progression of TNBC, we initially examined the expression levels of C9orf142 in two normal mammary epithelial cell lines as well as seven representative TNBC cell lines. The findings indicated that C9orf142 exhibited significant up‐regulation in TNBC cell lines when compared to normal cell lines (Figure 2A). Based on the relative expression levels of C9orf142 in these cell lines and their tumourigenic and metastatic properties, we stably overexpressed Flag‐C9orf142 in cells with low expression of C9orf142 (Figure 2B), whereas knocked down endogenous C9orf142 in cells with high expression of C9orf142 by lentiviral infection, respectively (Figure 2C). Results of in vitro functional experiments demonstrated that overexpression of C9orf142 in SUM159 and MDA‐MB‐231 cells enhanced cell growth (Figure 2D) and generated a greater number of viable colonies (Figure 2E,F). Conversely, the reduction of cell viability (Figure 2G) and colony formation capacity (Figure 2H,I) was observed when C9orf142 was knocked down using shRNA. Additionally, flow cytometry assays revealed that depletion of C9orf142 led to a rise in cells within the G1 phase, coupled with a decline in cells within the S phase (Figure 2J).

To further determine whether C9orf142 enhances the tumourigenic ability of TNBC cells in animal models, LM2‐4175 cells expressing shNC empty vector and shC9orf142 (#1 and #2) were injected into the mammary fat pads of female BALB/c nude mice (*n* = 10) (Figure 2K). Consistent with in vitro results, knockdown of C9orf142 significantly reduced tumour volume (Figure 2L, M) and weight (Figure 2N). The expression levels of C9orf142 in xenograft tumours were basically consistent with its in vitro validation results in cell lines (Figure S2A). Collectively, these results imply that C9orf142 enhances TNBC growth both in vitro and in vivo.

### 3.3 C9orf142 promotes the metastatic potential of TNBC cells

**FIGURE 3:**
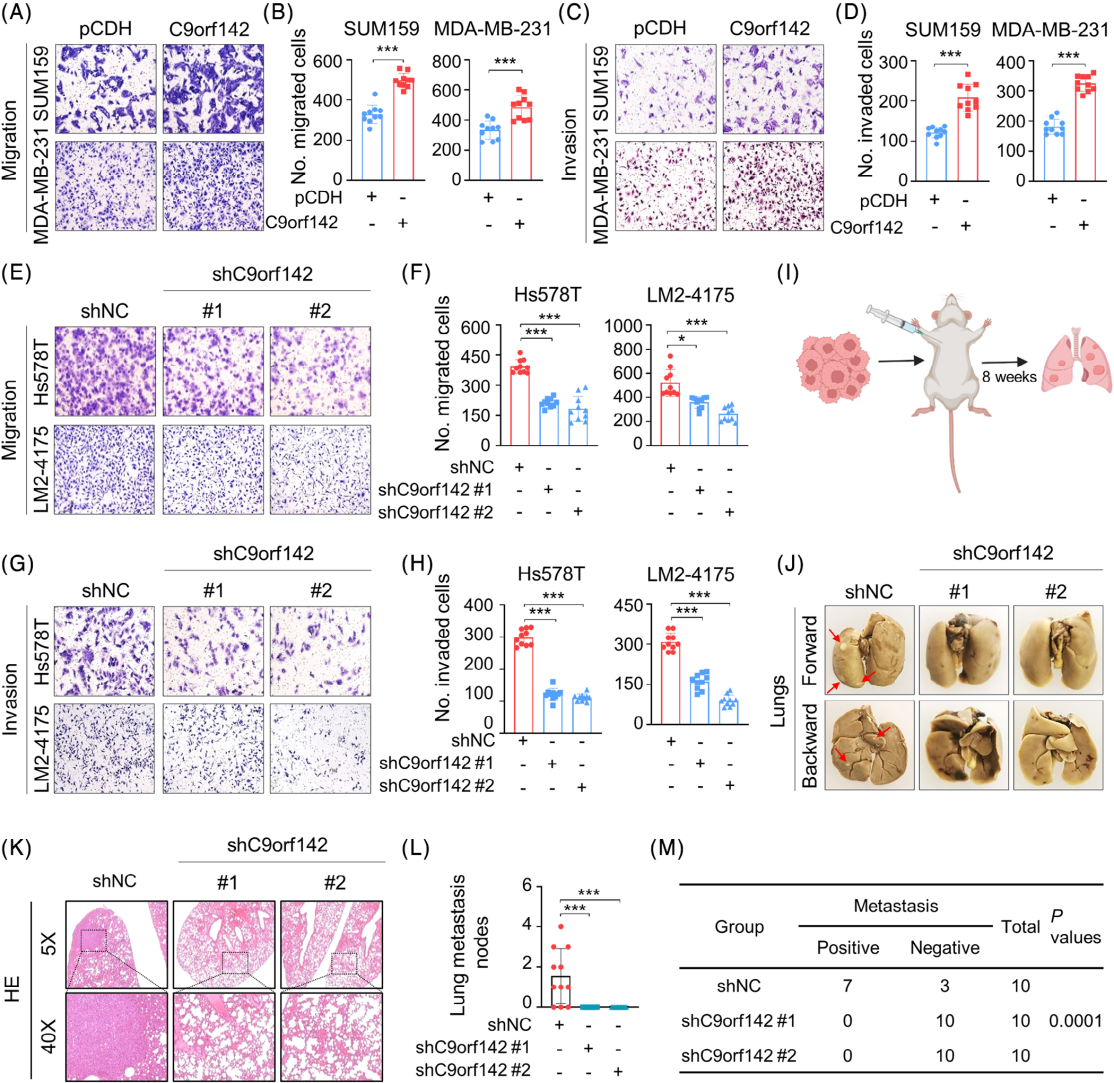
C9orf142 promotes migratory and invasive potential of TNBC cells in vitro and lung metastatic potential in vivo. (A–D) SUM159 and MDA‐MB‐231 cells stably expressing empty vector pCDH or Flag‐C9orf142 were subjected to Transwell migration assays (A and B) and Matrigel‐coated invasion assays (C and D). Representative images of migrated and invaded cells are shown in A and C, and corresponding quantitative results are shown in B and D, respectively. (E–H) Hs578T and LM2‐4175 cells stably expressing empty vector shNC or shC9orf142 (#1 and #2) were subjected to Transwell migration assays (E and F) and Matrigel‐coated invasion assays (G and H). Representative images of migrated and invaded cells are shown in E and G, and corresponding quantitative results are shown in F and H, respectively. (I–M) A total of 5 × 10^5^ LM2‐4175 cells stably expressing shNC or shC9orf142 (#1 and #2) were inoculated into mammary fat pads of 6‐week‐old BALB/c female nude mice (*n* = 10). After 8 weeks of injection, mice were sacrificed and lungs were removed. Schematic diagram depicting experimental procedure (I), representative images of lung metastasis (J), representative images of HE (hematoxylin‐eosin) staining of lung tissues (K), the number of metastasized nodes in the lungs (L) and the incidence of lung metastasis (M) are shown, respectively. Panel 3I was created with BioRender.com. *p < 0.05; **p < 0.01; ***p < 0.001; ns, no significance.

Due to the highly invasive and metastatic potential of TNBC, we then investigated the impact of C9orf142 on the metastatic capacities of these cells. The results showed that overexpression of C9orf142 increased the migratory and invasive potential of TNBC cells (Figure 3A–D). Conversely, the opposite effects were observed in cells with C9orf142 knockdown (Figure 3E–H).

To validate these results in a living organism, LM2‐4175 that were genetically modified to express shNC and shC9orf142 (#1 and #2) were injected into the mammary fat pads of female BALB/c nude mice (*n* = 10) for 8 weeks to generate the spontaneous metastatic models (Figure 3I). As expected, knockdown of C9orf142 dramatically reduced the quantity of metastatic lung nodes in the mice, the incidence of lung metastasis and the weight of the lungs (Figure 3J–M, and Figure S2B). Additionally, experimental murine model of axillary lymph node metastasis reconfirmed that C9orf142 knockdown dramatically reduced the occurrence of axillary lymph node metastasis, which was confirmed by the HE staining (Figure S2C,E). Collectively, these results suggest that C9orf142 boosts the invasive and metastatic potential of TNBC cells.

### 3.4 C9orf142 transcriptionally activates MTBP expression

**FIGURE 4:**
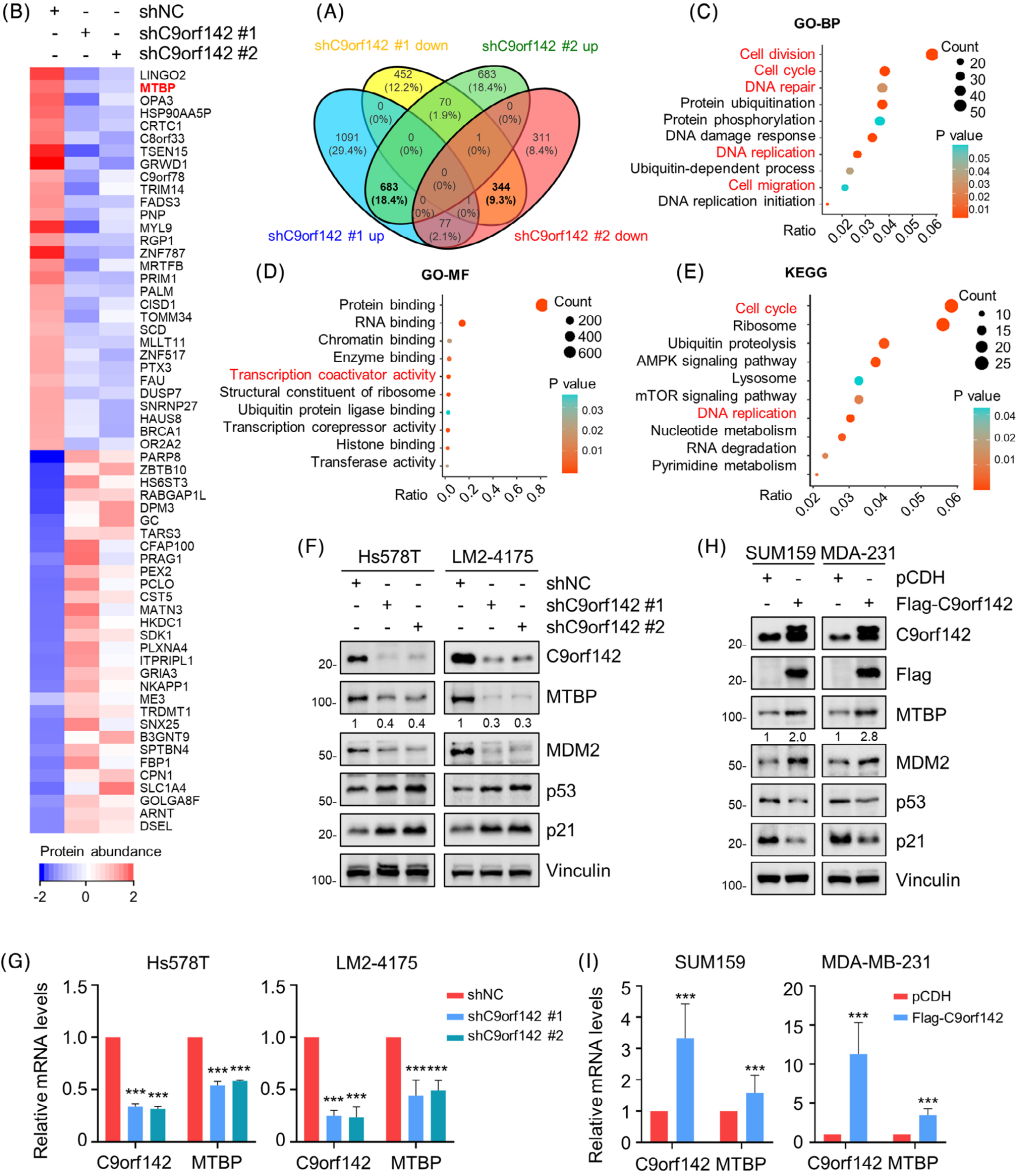
C9orf142 transcriptionally regulates MTBP expression. (A) LM2‐4175 cells stably expressing shNC and shC9orf142 (#1 and #2) were subjected to label‐free quantitative proteomic analysis. The numbers of differentially expressed proteins between cells expressing shNC and shC9orf142 based on the cut‐off value of 1.5‐fold change are shown. (B) Heatmap of the top 30 up‐regulated and down‐regulated proteins in LM2‐4175 cells stably expressing shNC and shC9orf142 (#1 and #2). (C and D) Gene ontology‐biological process (GO‐BP) (C) and GO‐molecular function (GO‐MF) (D) of differentially expressed proteins between cells expressing shNC and shC9orf142. (E) KEGG pathway analysis of differentially expressed proteins between cells expressing shNC and shC9orf142. (F and G) Hs578T and LM2‐4175 cells stably expressing shNC and shC9orf142 (#1 and #2) were subjected to immunoblotting assays with the indicated antibodies (F) and RT‐qPCR analysis (G). (H and I) SUM159 and MDA‐MB‐231 (MDA‐231) cells stably expressing pCDH and Flag‐C9orf142 were subjected to immunoblotting assays with the indicated antibodies (H) and RT‐qPCR analysis (I). *p < 0.05; **p < 0.01; ***p < 0.001; ns, no significance.

To investigate the underlying molecular mechanism of C9orf142 that promotes TNBC progression, we conducted label‐free quantitative proteomic assays. According to the cut‐off value of 1.5‐fold change, a total of 683 proteins were found to be upregulated and 344 proteins were down‐regulated following C9orf142 knockdown (Figure 4A), and the top 30 up‐regulated and down‐regulated proteins following C9orf142 knockdown are presented in Figure 4B. In order to acquire a comprehensive understanding of the biological roles of these proteins that were expressed differentialy, we conducted gene ontology‐biological process (GO‐BP) and GO‐molecular function (GO‐MF) analysis. It was found that these differentially expressed proteins were primarily participated in the following biological processes, such as cell division, cell cycle, cell migration, DNA repair and replication (Figures 4C). Molecular functions of these proteins were primarily related to protein/RNA/chromatin/enzyme binding, and transcriptional coactivator activity (Figure 4D). Additionally, Kyoto Encyclopedia of Genes and Genomes (KEGG) pathway analysis revealed that these proteins were mainly concentrated in cell cycle, DNA replication and ubiquitin proteolysis pathways (Figure 4E).

Given that the functional role of the top one down‐regulated protein LINGO2 (leucine rich repeat and Ig domain containing 2) following C9orf142 knockdown in human cancer is ambiguous, we selected MTBP, the second down‐regulated protein following C9orf142 knockdown, for further verification. Immunoblotting and RT‐qPCR analyses confirmed that knockdown of C9orf142 in Hs578T and LM2‐4175 cells resulted in a decrease in the protein and mRNA expression levels of MTBP (Figure 4F,G), whereas the opposite results were obtained in cells with overexpression of C9orf142 (Figure 4H,I). Furthermore, immunofluorescent staining assays also demonstrated a significant decrease in the expression levels of MTBP following C9orf142 knockdown (Figure S3A). These results suggest that C9orf142 may transcriptionally activate MTBP expression.

Previous studies have shown that MTBP enhances the stability of MDM2 and facilitates MDM2‐induced degradation of p53.^25^ In addition, p53 directly up‐regulates the expression levels of cyclin‐dependent kinase inhibitor p21, thereby arresting cells in the G1 phase to slow malignant progression.^35^, ^36^ Indeed, immunoblotting assays demonstrated that C9orf142 had a positive regulatory effect on the expression levels of MTBP and MDM2, while it had a negative regulatory effect on the expression levels of p53 and p21 (Figure 4F,H). These results collectively indicate that C9orf142 activates MTBP/MDM2/p53/p21 signaling axis in TNBC cells.

### 3.5 C9orf142 is enlisted to the MTBP promoter and boosts its promoter activities

**FIGURE 5:**
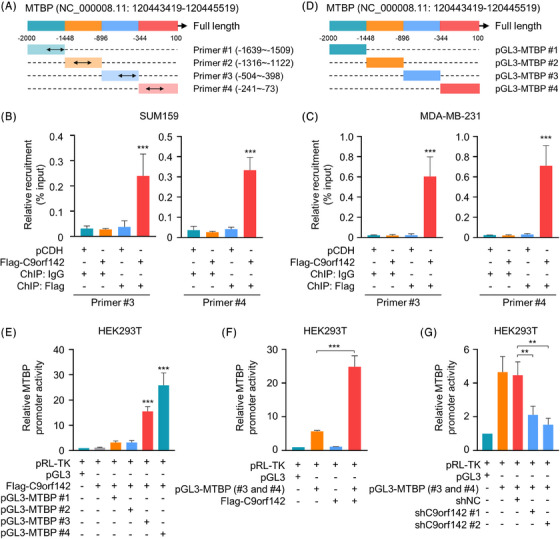
C9orf142 is recruited to the MTBP promoter and enhances its promoter activity. (A) Line diagram showing the regions of the MTBP promoter used for ChIP‐qPCR assays. (B and C) SUM159 and MDA‐MB‐231 cells stably expressing empty vector pCDH or Flag‐C9orf142 were subjected to ChIP assays and followed by RT‐qPCR assays (primer #3 and #4). The ChIP assays were carried out using an anti‐Flag antibody or IgG, where IgG was used as a negative control. Recruitment of Flag‐C9orf142 to the MTBP promoter was normalized to Input. Representative results of primer #1 and #2 are shown in Figure S3B and S3C. (D) Line diagram showing the regions of the MTBP promoter used for dual‐luciferase reporter assays. (E) HEK293T cells stably expressing pCDH or Flag‐C9orf142 were transfected with a luciferase reporter construct encoding pGL3 or pGL3‐MTBP (#1, #2, #3 and #4, respectively). Relative fluorescence activity was normalized to co‐transfected Renilla luciferase. (F) HEK293T cells stably expressing pCDH or Flag‐C9orf142 were transfected with a luciferase reporter construct encoding pGL3 or pGL3‐MTBP (#3 and #4 in combination). Relative fluorescence activity was normalized to co‐transfected Renilla luciferase. (G) HEK293T cells stably expressing shNC or shC9orf142 (#1 and #2) were transfected with a luciferase reporter construct encoding pGL3 or pGL3‐MTBP (#3 and #4 in combination). Relative fluorescence activity was normalized to co‐transfected Renilla luciferase. *p < 0.05; **p < 0.01; ***p < 0.001; ns, no significance.

To examine whether C9orf142 is recruited to the MTBP promoter, we performed immunoprecipitation using formaldehyde crosslinked chromatin (ChIP) from TNBC cells stably expressing empty vectors pCDH or Flag‐C9orf142. Subsequent RT‐qPCR tests were conducted with primers specifically designed for approximately every 500 base pair (bp) segment of the MTBP promoter (−2000 to +100) (Figure 5A). The findings indicated that C9orf142 was enlisted in two sections (−504 to −398 and −241 to −73) of the MTBP promoter (Figure 5B,C, Figure S3B,C). In order to investigate the impact of C9orf142 on MTBP promoter activities, about every 500 bp region of MTBP promoter (−2000 to +100) was amplified. These segments were then subcloned into the pGL3 vector, respectively, and transfected into HEK293T cells alone or in combination with Flag‐C9orf142 (Figure 5D). As shown in Figure 5E,F, C9orf142 enhanced MTBP promoter activities at those two regions (#3 and #4). Conversely, knockdown of C9orf142 reduced MTBP promoter activities (Figure 5G). Collectively, these results demonstrated that C9orf142 is enlisted to the MTBP promoter and boosts its promoter activities.

### 3.6 C9orf142 augments TNBC progression partially through regulating MTBP

**FIGURE 6:**
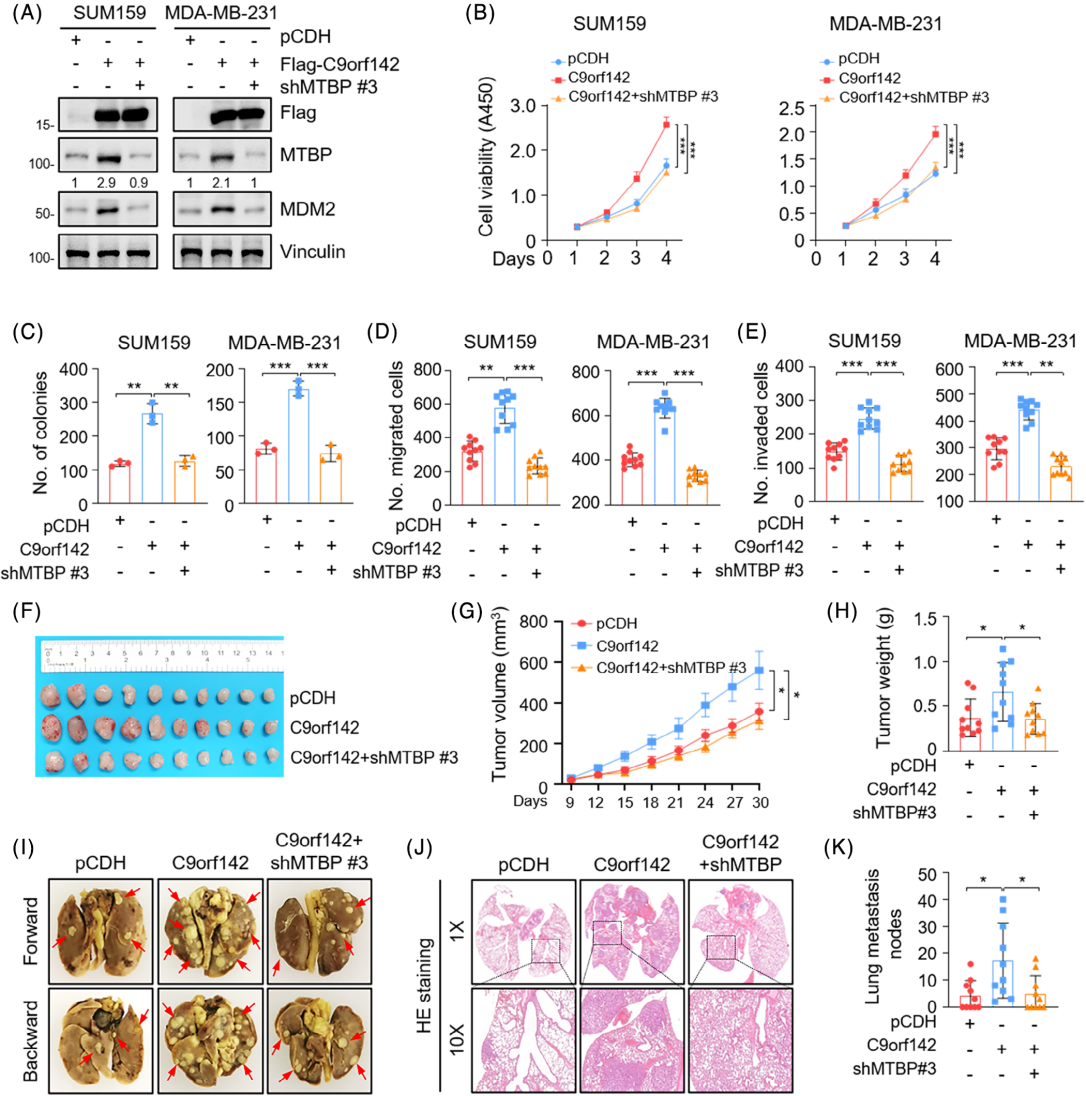
C9orf142 accelerates TNBC progression via regulating MTBP expression. (A) SUM159 and MDA‐MB‐231 cells stably expressing pCDH or Flag‐C9orf142 were transfected with shNC or shMTBP #3. After 48 h of transfection, cells were subjected to immunoblotting analysis with the indicated antibodies. (B and C) SUM159 and MDA‐MB‐231 cells stably expressing pCDH or Flag‐C9orf142 alone or in combination with shNC or shMTBP #3 were subjected to CCK‐8 (B) and colony formation assays (C). Representative images of survival colonies are shown inFigure S4B. (D and E) SUM159 and MDA‐MB‐231 cells stably expressing pCDH or Flag‐C9orf142 alone or in combination with shNC or shMTBP #3 were subjected to Transwell migration assays (D) and Matrigel‐coated invasion assays (E). Representative images of migrated and invaded cells are shown in Figure S4C andS4D. (F–H) A total of 3 × 10^6^ MDA‐MB‐231 cells stably expressing expressing pCDH or Flag‐C9orf142 alone or in combination with shNC or shMTBP #3 were inoculated into mammary fat pads of 6‐week‐old BALB/c female nude mice (*n* = 10). After 30 days of injection, mice were sacrificed and xenograft tumours were removed. Image of removed xenograft tumours (F), tumour volume (G), and tumour weight (H) are shown. (I‐K) A total of 1×10^6^ LM2‐4175 cells stably expressing shNC or shC9orf142 (#1 and #2) were injected into the tail vein of mammary fat pads of 7‐week‐old BALB/c female nude mice (*n* = 10). After 6 weeks of injection, mice were sacrificed and lungs were removed. Representative images of lung metastasis (I), representative images of HE staining of lung tissues (J), and the incidence of lung metastasis (K) are shown. *p < 0.05; **p < 0.01; ***p < 0.001; ns, no significance.

To verify whether C9orf142 promotes TNBC progression through regulating MTBP, we knocked down MTBP in TNBC cells with overexpression of Flag‐C9orf142. Immunoblotting assays reconfirmed that overexpression of C9orf142 resulted in an increase in the expression levels of MTBP and MDM2, whereas knockdown of MTBP partially abrogated these effects caused by overexpression of C9orf142 (Figure 6A and Figure S4A). Functional assays showed that knockdown of MTBP impaired the growth of TNBC cells (Figure 6B) and formation of colonies (Figure 6C and Figure S4B) triggered by ectopic expression of C9orf142. Similarly, the results revealed that the enhanced migration and invasion capacity of TNBC cells, which resulted from the overexpression of C9orf142, was partially attenuated following knockdown of endogenous MTBP (Figure 6D,E, Figure S4C,D). In the xenograft tumour mouse models, the C9orf142‐mediated enhancement of tumour growth was partially impaired after MTBP knockdown (Figure 6F–H). Furthermore, in the experimental lung metastasis models, the C9orf142‐mediated increase in the quantity of metastatic lung nodes and the occurrence of lung metastasis was compromised following knockdown of MTBP (Figure 6I–K, Figure S4E,F). In summary, these results indicate that C9orf142 contributes to TNBC progression by, at least partially, facilitating the transcriptional transactivation of MTBP.

### 3.7 C9orf142 promotes resistance of TNBC cells to CDK4/6 inhibitor abemaciclib

**FIGURE 7:**
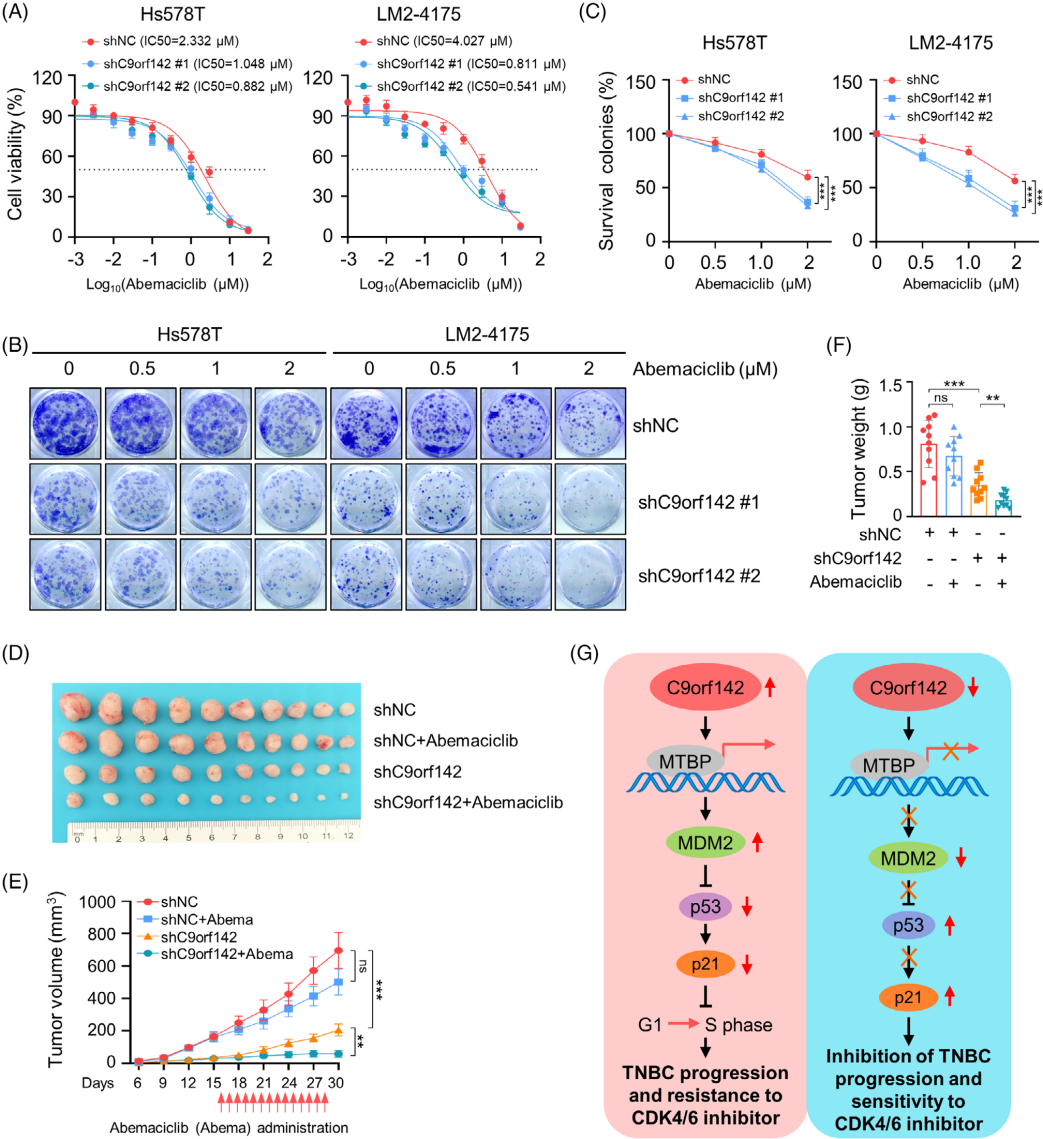
Knockdown of C9orf142 enhances the sensitivity of TNBC cells to abemaciclib both in vitro and in vivo. (A) Hs578T and LM2‐4175 stably expressing empty vector shNC or shC9orf142 (#1 and #2) were treated with or without increasing doses of abemaciclib. Cell viability was determined using CCK‐8 assays. The IC50 values are shown. (B and C) Hs578T and LM2‐4175 cells stably expressing empty vector shNC or shC9orf142 (#1 and #2) were treated without or with the indicated concentrations of abemaciclib and subjected to colony formation assays. Representative images of survival colonies and corresponding quantitative results are shown in B and C, respectively. (D–F) A total of 1 × 10^6^ LM2‐4175 cells stably expressing shNC or shC9orf142 (#1 and #2) were inoculated into mammary fat pads of 6‐week‐old BALB/c female nude mice (*n* = 20). After 16 days of injection, each group is randomly divided into two groups (*n* = 10), and the mice were administered with or without abemaciclib (25 mg/kg) by oral gavage once daily for 14 consecutive days. After 30 days of injection, mice were sacrificed and xenograft tumours were removed. Image of removed xenograft tumours (D), tumour volume (E) and tumour weight (F) are shown. (G) The proposed working model. C9orf142 transcriptionally activates MTBP and regulates its downstream MDM2/p53/p21 signaling axis and cell cycle transition from G1‐to‐S phase to drive the progression and resistance to CDK4/6 inhibitor in TNBC. *p < 0.05; **p < 0.01; ***p < 0.001; ns, no significance.

The mechanism of CDK4/6 inhibitors against cancer is to prevent the transition of cell cycle from G1 phase to S phase.^37^ Our above results demonstrated that C9orf142 had a positive effect on the expression of MTBP and MDM2, while it had a negative effect on the expression of p53 and p21 (Figures 4F and 4H), thus promoting transition of cell cycle through G1‐to‐S phase. Flow cytometry assays also demonstrated that depletion of C9orf142 led to an increase in cells in the G1 phase, along with a decrease of cells in the S phase (Figure 2J). Therefore, we proceeded to examine the influence of C9orf142 on the sensitivity of TNBC cells to CDK4/6 inhibitor abemaciclib. We found that C9orf142‐depleted cells were more sensitive to abemaciclib compared to control cells (Figure 7A). Similarly, the colony formation assays yielded comparable outcomes (Figure 7B,C). Moreover, in xenograft tumour models, the tumour growth inhibitory effects (including tumour volume and weight in mice) caused by C9orf142 knockdown were significantly greater than that observed in control mice following abemaciclib administration (Figures 7D–7F). Collectively, these results indicate that C9orf142 promotes resistance of TNBC cells to the CDK4/6 inhibitor abemaciclib.

## 4 DISCUSSION

This study presents several noteworthy findings regarding the previously underestimated functional and mechanistic roles of C9orf142 in the advancement and resistance to CDK4/6 inhibitors in TNBC (Figure 7G).

C9orf142, an extensively preserved member of the XRCC4 family and a novel component of the NHEJ complex, exhibits broad expression in all human tissues and assumes a pivotal role in the DNA damage repair.^13^ Mechanistically, C9orf142 shares a comparable structure and function to its paralogues XRCC4 and XLF. C9orf142 and its paralogues form a stable NHEJ complex to repair DNA breaks, thus enhancing cellular resistance to DNA double strand breaks (DSB)‐inducing agents, such as VP16, doxorubicin and irradiation.^13^ In addition, very limited evidence indicates the potential involvement of C9orf142 in cancer progression and development of resistance to therapy. For example, C9orf142 is up‐regulated in colon cancer^19^ and in cisplatin and doxorubicin resistant osteosarcoma cells.^20^ Inhibition of C9orf142 provides a promising way to overcome doxorubicin and cisplatin resistance in osteosarcoma,^20^ and temozolomide resistance in glioma.^21^ However, the biological function of C9orf142 in breast cancer has never been investigated. This study presents the initial proof that C9orf142 exhibited a notable increase in TNBC patients and those with lymph node metastasis, and its elevation was associated with unfavorable clinical outcome (Figure 1). The experiments of gain‐ and loss‐of functions demonstrated that C9orf142 accelerated TNBC growth and metastasis (Figures 2 and 3), suggesting that C9orf142 is a newly identified oncoprotein in TNBC.

MTBP, a binding partner of MDM2, is involved in tumourigenesis and metastasis.^24^, ^38^ Previous studies have demonstrated that MTBP is frequently elevated in multiple types of human cancer, such as breast ductal carcinoma, cervical carcinoma, colorectal carcinoma, gastric adenocarcinoma, glioblastoma, lung cancer and prostate carcinoma,^29^ and contributes to malignant phenotypes of breast cancer,^28^ lung cancer,^39^ glioblastoma,^30^ and colon cancer.^40^ Significantly, analysis of the TCGA dataset revealed that MTBP is amplified in 19% breast cancer, and its mRNA expression levels are notably elevated in TNBC relative to those in other subtypes of breast cancer.^28^ Despite these observations, the regulatory mechanism for MTBP in human cancer has not yet been fully elucidated. In this study, it was discovered that MTBP is a downstream transcriptional target of C9orf142 (Figures 4 and 5). In this context, C9orf142 is enlisted to the promoter of MTBP and boosts its promoter activities.

MDM2, an E3 ubiquitin‐protein ligase and also known as oncoprotein, is critically involved in the advancement of cancer and the development of therapeutic resistance of chemotherapy (such as cisplatin, doxorubicin, gemcitabine and 5‐FU), radiotherapy, targeted agents (such as tyrosine kinase inhibitors).^41^ Previous studies have indicated that MTBP contributes to tumour progression and therapeutic sensitivity through stabilizing MDM2, thereby promoting MDM2‐mediated degradation of p53 and downregulating p53 target gene p21.^30^ Consistently, we observed that C9orf142 has a positive regulatory effect on the levels of MTBP and MDM2, while having a negative regulatory effect on the levels of p53 and p21 (Figure 4). Significantly, depletion of MTBP hindered cell proliferation, migration, and invasion induced by overexpressing C9orf142, as well as suppressed tumour growth and lung metastasis in mice (Figure 6). These results indicate that C9orf142 boosts the advancement of TNBC through, at least in part, regulating MTBP/MDM2/p53/p21 axis.

Given the significant roles of MTBP/MDM2/p53/p21 signaling axis in regulating G1 checkpoint of the cell cycle in human cancers^24^, ^28^, ^30^ and that C9orf142 deficiency led to G1 phase accumulation of TNBC cells (Figure 2J), we explored the effects of depletion of C9orf142 on the sensitivity of TNBC cells to CDK4/6 inhibitor abemaciclib. CDK4/6 inhibitors are not priority treatment strategies for TNBC patients due to their high frequency of RB1 loss/mutations and upregulation of CDK6 and cyclin A2.^44^ However, recently, evidence from several clinical trials demonstrate that some TNBC patients can benefit from CDK4/6 inhibitor treatment because of the heterogeneity of TNBC.^5^, ^45^ The mechanism of CDK4/6 inhibitors targeting cancer is to prevent the transition from G1‐phase to S‐phase of the cell cycle.^46^ Our results indicated that C9orf142 had a positive effect on the expression of MTBP and MDM2, while it had a negative effect on the expression of p53 and p21, thereby promoting the transition of the cell cycle from G1 phase to S phase. Flow cytometry analysis also confirmed that the absence of C9orf142 resulted in an increase in G1 phase cells and a decrease in S phase cells. Abemaciclib is the strongest CDK4/6 inhibitor in terms of CDK4/6 enzyme inhibition potential. Therefore, we continued to investigate the effect of C9orf142 on the sensitivity of TNBC cells to the CDK4/6 inhibitor abemaciclib. The noteworthy discovery of this study is that suppressing C9orf142 remarkably enhanced TNBC cellular sensitivity to CDK4/6 inhibitor abemaciclib. Therefore, these findings imply that C9orf142 serves as a potential biomarker for CDK4/6 inhibitor resistance in TNBC.

Taken together, findings presented here suggest that C9orf142 acts as a transcriptional activator of MTBP to regulate the MDM2/p53/p21 signaling axis and drives progression and resistance to CDK4/6 inhibitors in TNBC. These results highlight that C9orf142 could function as a potential therapeutic targe for TNBC patients.

## CONFLICT OF INTEREST STATEMENT

The authors declare no potential conflicts of interest.

## Supporting information

Supporting Information

## Data Availability

The results shown here are based, in part, on data generated by the FUSCC‐TNBC cohort and TCGA are available in a public repository from the www.cbioportal.org/ websites. All data needed to evaluate the conclusions in the paper are present in the paper and/or the Supplementary Materials.
