# Supplementary material for: C9orf142 transcriptionally activates MTBP to drive progression and resistance to CDK4/6 inhibitor in triple‐negative breast cancer
Source: Clin Transl Med. 2023 Nov 27;13(11):e1480. doi: 10.1002/ctm2.1480 (PMC10679971; doi:10.1002/ctm2.1480)
Supplement: Supplementary file 1 — Supporting Information [file CTM2-13-e1480-s001.docx]

**Supplementary information for**

Liao et al. C9orf142 transcriptionally activates MTBP to drive progression and resistance to CDK4/6 inhibitor in triple-negative breast cancer

**Supplementary information contains:**

Supplementary Figures 1-4

Supplementary Tables 1-5

**Supplementary figures and figure legends**

**
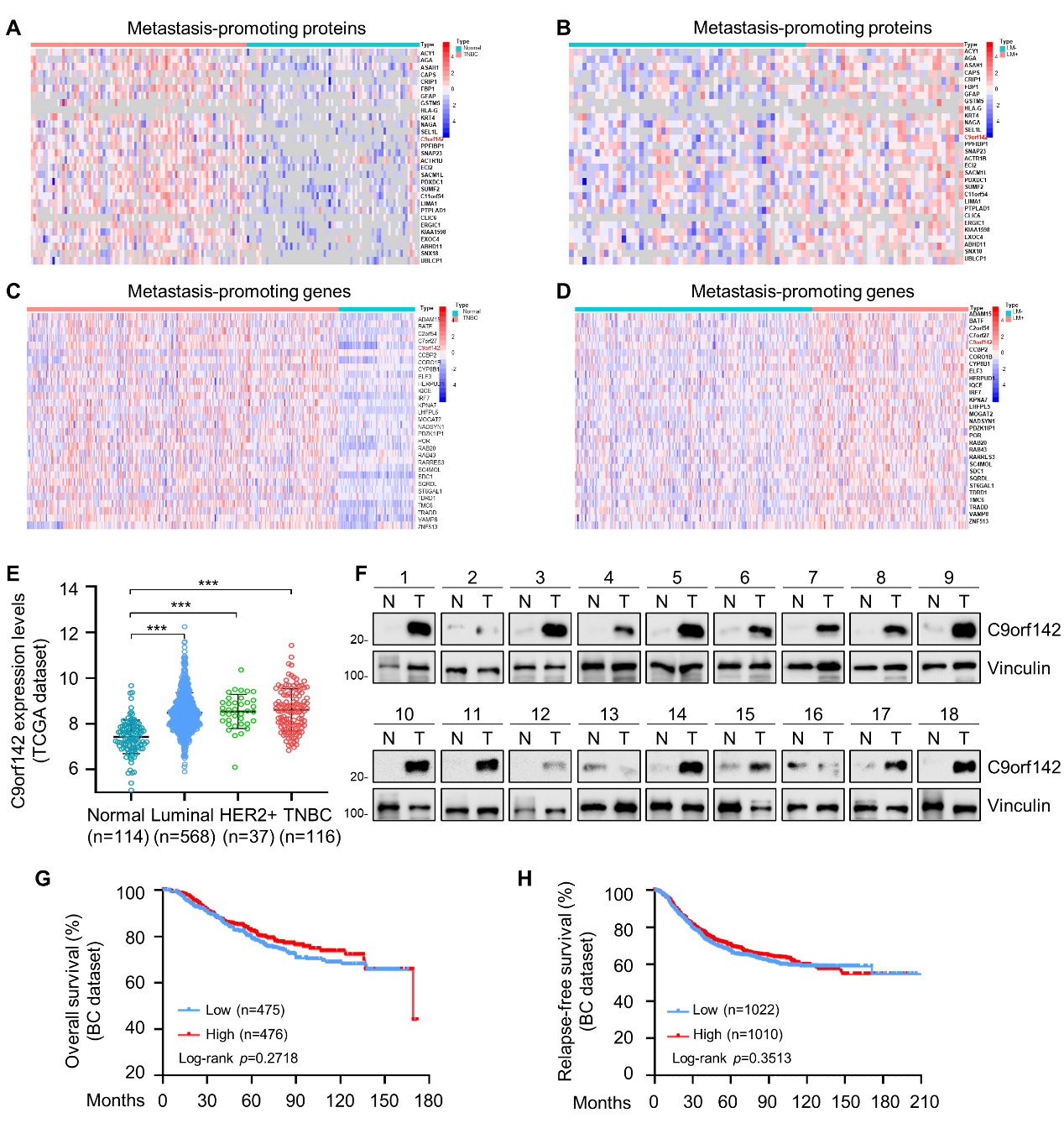
**

**Figure S1. C9orf142 is aberrantly up-regulated in TNBC tissues**

**(A)** The top 30 metastatic-promoting oncoproteins in the FUSCC-TNBC proteomic database (TNBC specimens: n= 90; adjacent normal specimens: n=72).

**(B)** The top 30 metastatic-promoting oncoproteins in the FUSCC-TNBC proteomic database (negative lymph node metastasis specimens: n= 54; positive lymph node metastasis specimens: n=36).

**(C)** The top 30 metastatic-promoting oncogenes in the FUSCC-TNBC RNA-seq database (TNBC specimens: n= 360; adjacent normal specimens: n=88).

**(D)** The top 30 metastatic-promoting oncogenes in the FUSCC-TNBC RNA-seq database (negative lymph node metastasis specimens: n= 216; positive lymph node metastasis specimens: n=144).

**(E)** Expression levels of C9orf142 in different breast cancer subtypes in the TCGA database.

**(F)** Immunoblotting analysis of C9orf142 protein expression levels in 18 pairs of TNBC tissues and matched normal breast tissues. Corresponding quantitative results are shown in Figure 1J.

**(G and H)** Kaplan–Meier analysis of the overall survival (G) and relapse-free survival (H) of all types of breast cancer patients with high or low expression levels of C9orf142 using Kaplan-Meier Plotter database (http://kmplot.com/analysis/index.php?p=service). *, p<0.05; **, p<0.01; ***, p<0.001; ns, no significance.

**
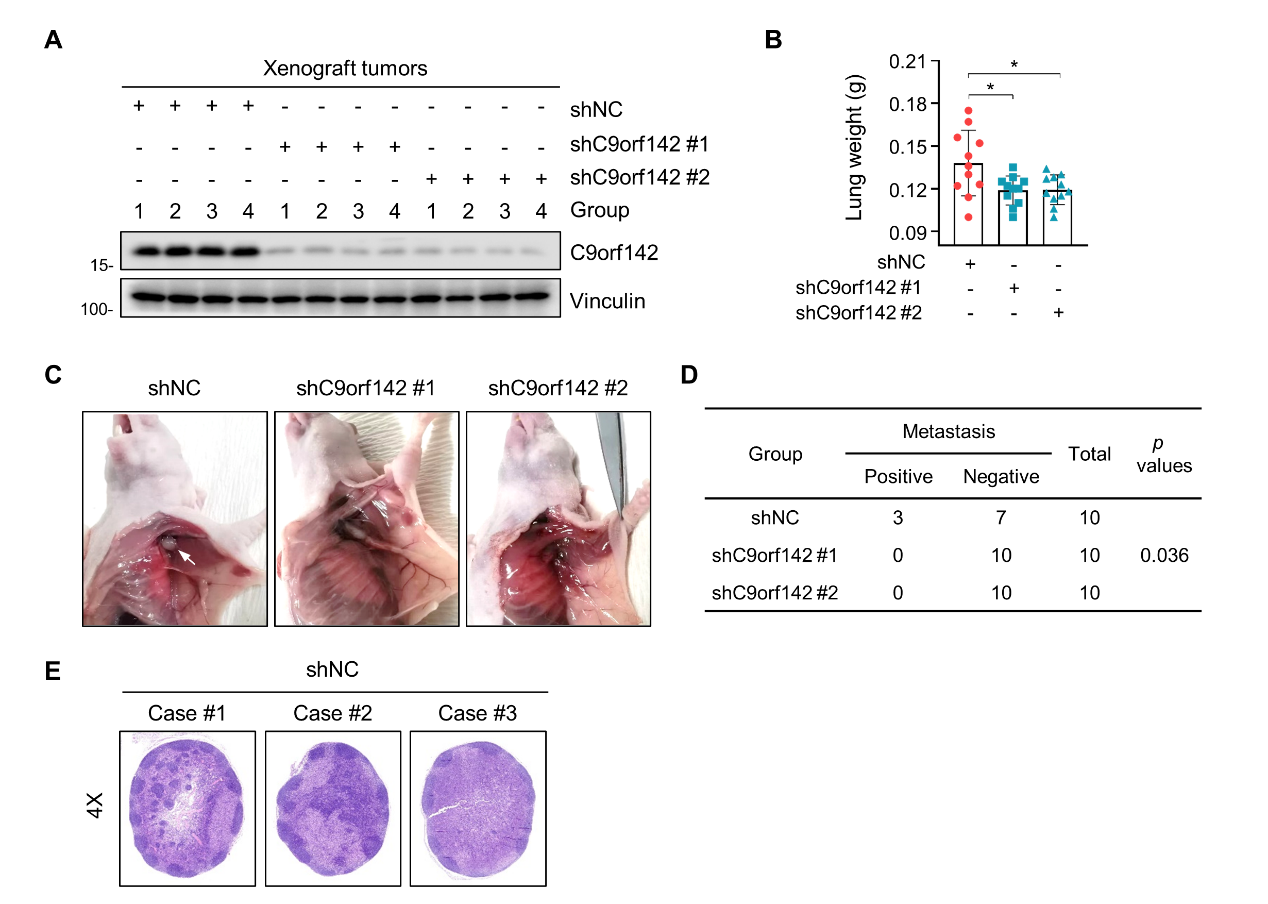
**

**Figure S2. C9orf142 promotes TNBC progression**

**(A)** Immunoblotting assays showing the expression levels of C9orf142 in xenograft tumor.

**(B)** A total of 5×10^5^ LM2-4175 cells stably expressing shNC or shC9orf142 (#1 and #2) were inoculated into mammary fat pad of 6-week-old BALB/c female nude mice (n=10). After 8 weeks of injection, mice were sacrificed and lungs were removed. The weight of the lungs is shown.

**(C-E)** A total of 1×10^6^ LM2-4175 cells stably expressing shNC or shC9orf142 (#1 and #2) were inoculated into mammary fat pad of 6-week-old BALB/c female nude mice (n=10). After 30 days of injection, mice were sacrificed and xenograft tumors were removed. Images of metastatic axillary lymph node (C), the incidence of axillary lymph node metastasis (D) and H-E staining of metastatic axillary lymph node (E) are shown.

**
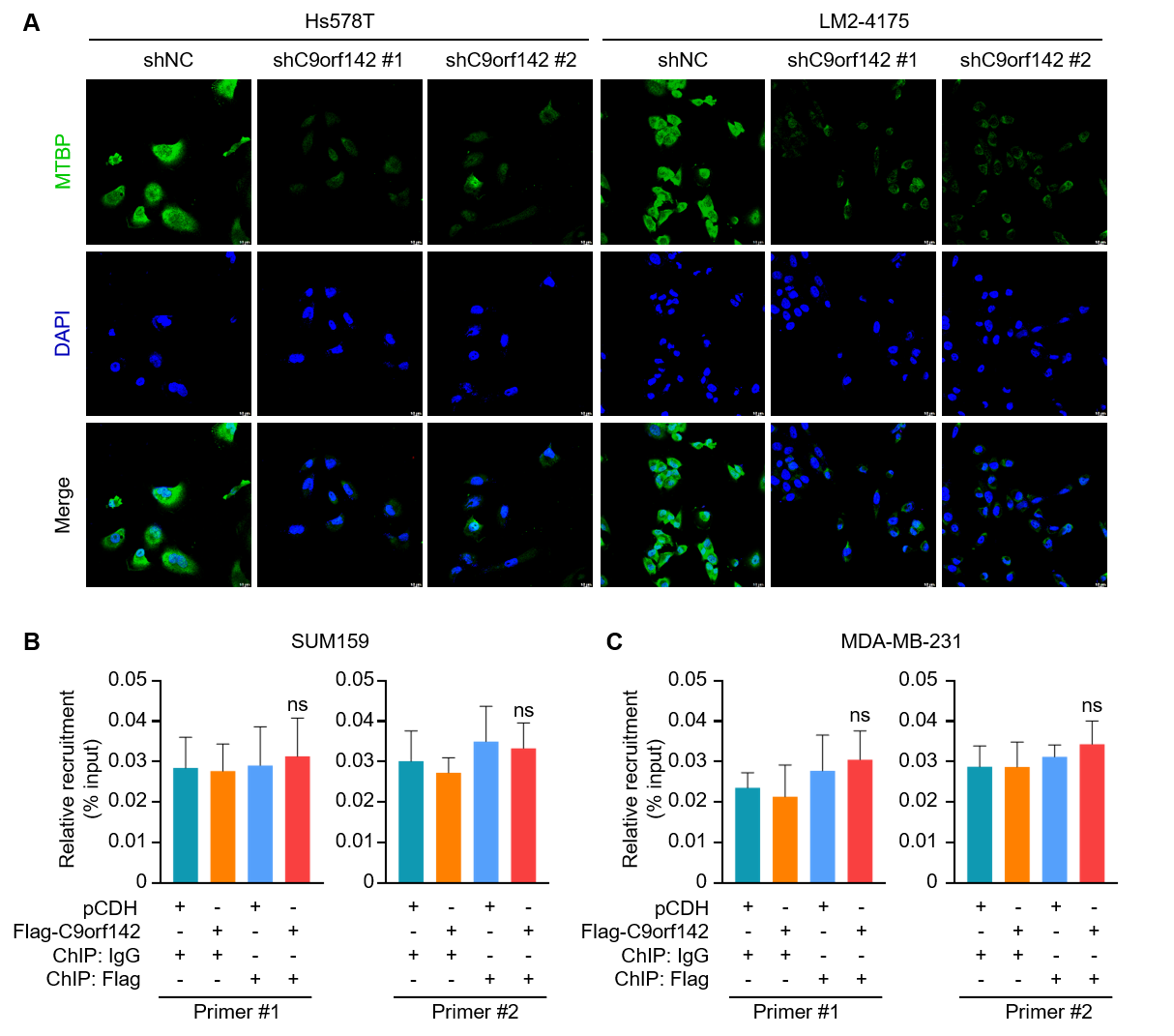
**

**Figure S3. C9orf142 is recruited to the MTBP promoter**

**(A)** Immunofluorescent staining of the expression of MTBP (green) in Hs578T and LM2-4175 cells stably expressing shNC and shC9orf142 (#1 and #2).

**(B and C)** SUM159 and MDA-MB-231 cells stably expressing empty vector pCDH or Flag-C9orf142 were subjected to ChIP assays and followed by qPCR assays (primer #1 and #2). The ChIP assays were carried out using an anti-Flag antibody or IgG as a negative control. Recruitment of Flag-C9orf142 to the MTBP promoter was normalized to Input. Representative results of primer #3 and #4 are shown in Figure. 5B and 5C.

**
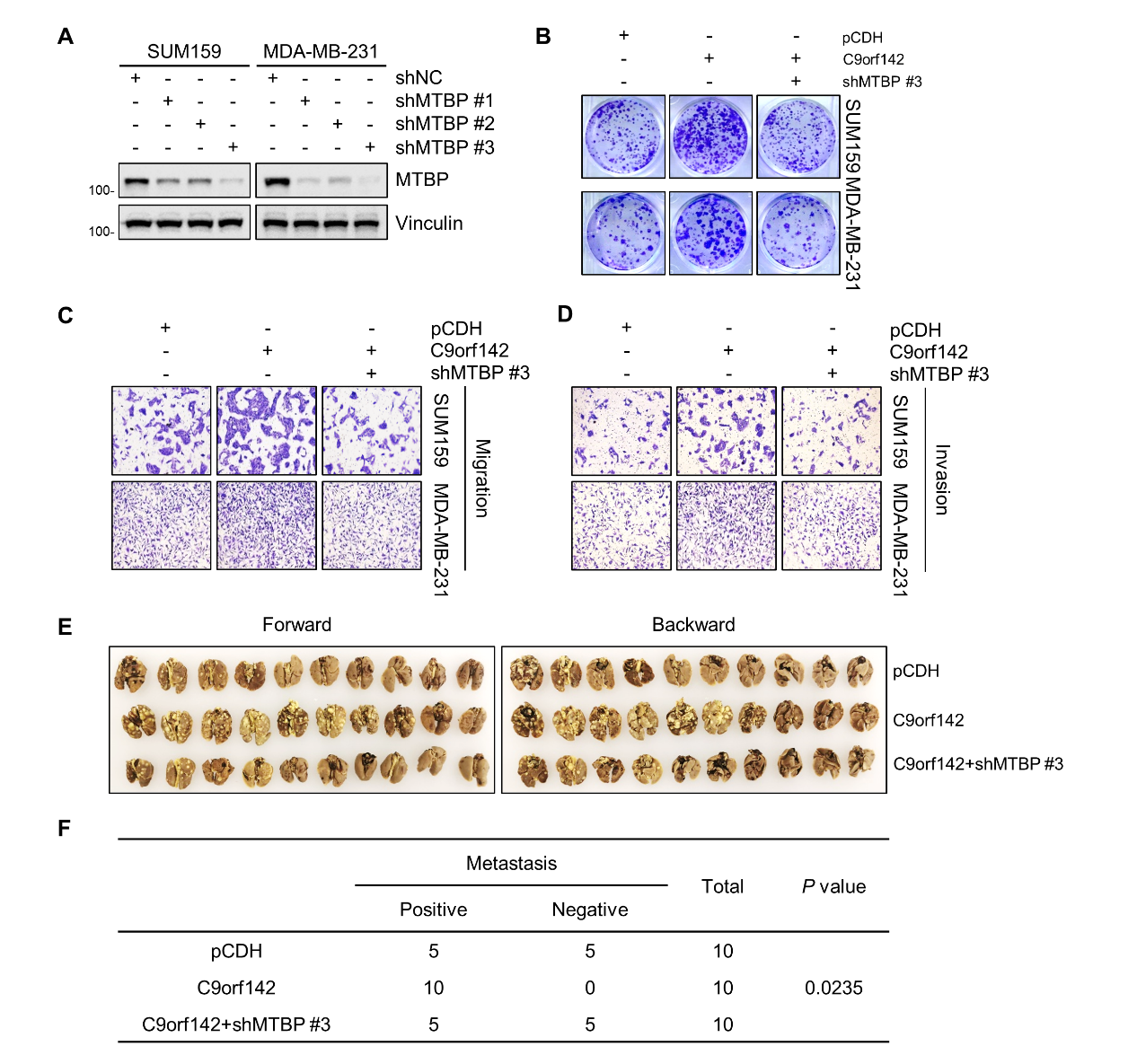
**

**Figure S4. C9orf142 accelerates TNBC progression via regulating MTBP expression**

**(A)** Hs578T and LM2-4175 cells stably expressing shNC and shMTBP (#1, #2 and #3) were subjected to immunoblotting analysis with the indicated antibodies.

**(B)** SUM159 and MDA-MB-231 cells stably expressing pCDH or Flag-C9orf142 alone or in combination with shNC or shMTBP #3 were subjected to colony formation assays. Corresponding quantitative results are shown in Figure. 6C.

**(C and D)** SUM159 and MDA-MB-231 cells stably expressing pCDH or Flag-C9orf142 alone or in combination with shNC or shMTBP #3 were subjected to Transwell migration assays (C) and Matrigel-coated invasion assays (D). Corresponding quantitative results are shown in Figures 6D and 6E, respectively.

**(E and F)** A total of 1×10^6^ LM2-4175 cells stably expressing shNC or shC9orf142 (#1 and #2) were injected into the tail vein of mammary fat pad of 7-week-old BALB/c female nude mice (n=10). After 6 weeks of injection, mice were sacrificed and lungs were removed. Images of removed lung tissues (E) and the incidence of lung metastasis (F) are shown.

**Supplementary tables**

**Table S1. Targeting sequences for shRNAs.**

| shRNAs | Primers | Sequences |
| --- | --- | --- |
| shC9orf142 #1 | Forward | CCGGCTCTTCTTACCAGACCCAGATCTCGAGATCTGGGTCTGGTAAGAAGAGTTTTTG |
|  | Reverse | AATTCAAAAACTCTTCTTACCAGACCCAGATCTCGAGATCTGGGTCTGGTAAGAAGAG |
| shC9orf142 #2 | Forward | CCGGACAGAGCATCCCTGACGCTTTCTCGAGAAAGCGTCAGGGATGCTCTGTTTTTTG |
|  | Reverse | AATTCAAAAAACAGAGCATCCCTGACGCTTTCTCGAGAAAGCGTCAGGGATGCTCTGT |
| shMTBP #1 | Forward | CCGGCAGTAATAGCAGGGAATCATTCTCGAGAATGATTCCCTGCTATTACTGTTTTTG |
|  | Reverse | AATTCAAAAACAGTAATAGCAGGGAATCATTCTCGAGAATGATTCCCTGCTATTACTG |
| shMTBP #2 | Forward | CCGGGCCATGTACCATTAGTAACATCTCGAGATGTTACTAATGGTACATGGCTTTTTG |
|  | Reverse | AATTCAAAAAGCCATGTACCATTAGTAACATCTCGAGATGTTACTAATGGTACATGGC |
| shMTBP #3 | Forward | CCGGCCCTGAAGAAACACAGTATTACTCGAGTAATACTGTGTTTCTTCAGGGTTTTTG |
|  | Reverse | AATTCAAAAACCCTGAAGAAACACAGTATTACTCGAGTAATACTGTGTTTCTTCAGGG |

**Table S2. Primers used for molecular cloning of expression vectors.**

| Plasmids | Primers | Sequences |
| --- | --- | --- |
| Flag-pCDH-C9orf142 | Forward | GATTCTAGAGCTAGCGAATTCGCCACCATGGATCCGCTGTCGCCGCCG |
|  | Reverse | ATGGTCTTTGTAGTCGGATCCGGTCTCATCGAAGTCCACGCCACCAGC |
| Flag-pLVX-C9orf142 | Forward | GGATCTATTTCCGGTGAATTCGCCACCATGGATCCGCTGTCGCCGCCG |
|  | Reverse | GGGATCCGCGGCCGCTCTAGATTACTTGTCATCGTCGTCCTTGTAATCGGTCTCATCGAAGTCCACGCCACCAGC |

**Table S3. Antibodies used in this study.**

| Antibodies | Vendors | Cat# | Host | Working concentration |
| --- | --- | --- | --- | --- |
| C9orf142 | CST | 92448S | Rabbit | 1:1000 (WB) |
| Vinculin | Sigma | V9131 | Mouse | 1:5000 (WB) |
| MTBP | Proteintech | 28356-1-AP | Rabbit | 1:1000 (WB) |
| MTBP | Santa Cruz | B-5 | Mouse | 1:50 (IF) |
| MDM2 | Santa Cruz | D-12 | Mouse | 1:500 (WB) |
| P53 | CST | 2527S | Rabbit | 1:1000 (WB) |
| P21 | CST | 2947S | Rabbit | 1:1000 (WB) |
| p-RB1 (Ser807/811) | CST | 8516T | Mouse | 1:1000 (WB) |
| RB1 | Abcam | ab181616 | Rabbit | 1:2000 (WB) |
| Cyclin A2 | Sinobiological | 201099-T46 | Rabbit | 1:1000 (WB) |
| CDK4 | Abcam | ab108357 | Rabbit | 1:2000 (WB) |
| CDK6 | Abcam | ab124821 | Rabbit | 1:2000 (WB) |

Note: WB, Western blot; IF, immunofluorescence;

**Table S4. Primers for qPCR analysis.**

| Genes | Primers | Sequences |
| --- | --- | --- |
| MTBP promoter #1 | Forward | GGAGTTCAATTCCTGGGGAGA |
|  | Reverse | GCTCTATTCCTGAGGCAACCTAA |
| MTBP promoter #2 | Forward | CCAGTTTTTGTCCCCATCCATTG |
|  | Reverse | GCCGCTCCAAGTATGGTTC |
| MTBP promoter #3 | Forward | GACTGACGACCCTCTTGTGC |
|  | Reverse | AAGGAGAGCGTTGGTTCCG |
| MTBP promoter #4 | Forward | AACACCCAAGCAGGACAGTT |
|  | Reverse | GCAGTTTTTCCACCAAGCGA |
| C9orf142 | Forward | TTCGTGTGCTACTGCGAAGG |
|  | Reverse | GTGAAGCAGGTGCTCCAAAG |
| MTBP | Forward | TCCTGTAGTTTCGTCAGATCCT |
|  | Reverse | CCGTTTCAATCGGGATACTTCA |
| GAPDH | Forward | TCGGAGTCAACGGATTTGGT |
|  | Reverse | TTCCCGTTCTCAGCCTTGAC |

**Table S5. Primers used for molecular cloning of pGL3-MTBP promoter vectors.**

| Genes | Primers | Sequences |
| --- | --- | --- |
| pGL3-MTBP #1 | Forward | CGAGCTCTTACGCGTGCTAGCAATGTCAACAATTTCTTTGAAAAATAAC |
|  | Reverse | ACTTAGATCGCAGATCTCGAGCTTTCTGGCCTAGAATTTTC |
| pGL3-MTBP #2 | Forward | CGAGCTCTTACGCGTGCTAGCTCTCCACCAGATGCTTTC |
|  | Reverse | ACTTAGATCGCAGATCTCGAGAGTGGTCAGGTTTTGTTTG |
| pGL3-MTBP #3 | Forward | CGAGCTCTTACGCGTGCTAGCTTCAACACACTCCATAGTATTTC |
|  | Reverse | ACTTAGATCGCAGATCTCGAGGTGAGCTCGGACTCTTATG |
| pGL3-MTBP #4 | Forward | CGAGCTCTTACGCGTGCTAGCGCCCTAGAGAAACTCGAC |
|  | Reverse | ACTTAGATCGCAGATCTCGAGAGATCACCAGCAGCAG |
